# Physical Activity Is Associated With Reduced Mild Depression and Altered Gut Microbiota in Japanese Adult Women

**DOI:** 10.7759/cureus.90104

**Published:** 2025-08-14

**Authors:** Hiroki Hamada, Eri Oyanagi, Chihiro Watanabe, Takafumi Aoki, Masato Kawashima, Hiroki Yajima, Takeshi Yoda, Michael J Kremenik, Toshihiro Takao, Hiromi Yano

**Affiliations:** 1 Department of Health and Sports Science, Kawasaki University of Medical Welfare, Kurashiki, JPN; 2 Department of Health Check-up Center, Kawasaki Medical School Hospital, Kurashiki, JPN; 3 Department of Clinical Nutrition, Kawasaki University of Medical Welfare, Kurashiki, JPN; 4 Department of Humanities and Social Sciences, Kyushu Institute of Technology, Kitakyushu, JPN; 5 Department of Public Health, Kawasaki Medical School Hospital, Kurashiki, JPN; 6 Department of Health Care Medicine, Kawasaki Medical School Hospital, Kurashiki, JPN

**Keywords:** bdi-ⅱ, depression, gut microbiota, holdemania, ipaq, physical activity

## Abstract

Background: Although there is much evidence for the influence of physical activity on mental illness, there is a lack of analysis of the relationship between physical activity and depression via gut microbiota. This study aimed to examine whether physical activity is associated with reduced mild depression and altered gut microbiota composition in Japanese adult women.

Methods: The participants answered the International Physical Activity Questionnaire (IPAQ) to assess physical activity and the Beck Depression Inventory-II (BDI-II) to assess symptoms of depression. The inclusion criteria were as follows: age 50 to 80 years, non-smokers, and not taking antibiotics at the time of study entry. The items measured were the gut microbiota, dietary questionnaire (Brief-Type Self-Administered Diet History Questionnaire (BDHQ)), body weight, body fat percentage, blood pressure, and blood test results (glucose, HbA1c, triglyceride, high-density lipoprotein (HDL)-cholesterol, and low-density lipoprotein (LDL)-cholesterol). Comparison of means between groups was analyzed using a two-way analysis of variance (ANOVA), and then, the Dunn test was conducted as a post hoc test.

Results: Alpha- and beta-diversities showed no significant differences among all groups. In the comparison between the Dep (-) group and the Dep (+) group, six bacteria were identified, whereas in the comparison between the Sed group and the Act group, five bacteria were extracted (p < 0.05). In particular, *Megamonas* showed a positive correlation with triglycerides and HbA1c, and *Holdemania*, which acts to suppress depression, was common in the Act and Dep (-) groups.

Conclusion: Our findings support the concept that physical activity influences brain functions in women with mild depression via the gut microbiota. This suggests that promoting physical activity that induces changes in the gut microbiota may be a protective approach to depression in women.

## Introduction

Mental illnesses affect approximately 13.9% of the world’s population, most commonly major depressive disorder (MDD), which affects about 2% of the global population [1]. MDD is characterized by loss of pleasure and interest, as well as persistent depressed mood.

Consequently, it is an impairment that has a significant negative impact on activities of daily living, quality of life, cognitive functioning, employment status, and work productivity [2]. Moreover, meta-analyses of depression and gender differences have shown that women have higher levels of severe depression and depressive symptoms than men [3].

The MDD has been found to be associated with gut microbiota, and three possible mechanisms of gut bacteria on the gut-brain correlation have been postulated: modification of autonomic/sensory-motor connections, immune activation, and modulation of neuroendocrine pathways [4]. In other words, a focus on the gut microbiota holds promise as one of the new perspectives for the treatment and improvement of MDD.

Previous studies have reported that changes in the gut microbiota through prebiotics and probiotics improve human mood and anxiety, and a study on Japanese participants reported that the gut microbiota may influence depressive symptoms through butyric acid, a short-chain fatty acid (SCFA) [5]. Interestingly, that study [5] suggested that *Holdemania*, *Lactobacillus*, and *Mitsuokella* may influence depressive status, as well as butyrate-producing bacteria. However, previous studies on gut microbiota and depressive symptoms are often contradictory and have not led to consistent conclusions.

On the other hand, habitual exercise is effective not only in preventing lifestyle-related diseases such as obesity, hypertension, and heart diseases but also in preventing mental disorders such as depression [6]. The involvement of the gut microbiota is thought to be a factor in the prevention of depression through exercise habits [7]. The relationship between exercise and gut microbiota composition is bidirectional. Namely, exercise intervention studies in humans have shown that regular exercise alters gut microbiota composition [8]. In addition, a previous study in mice showed that spontaneous exercise changes the bacteria associated with depression-like behavior; decreases in *Turicibacter*, *Allobaculum*, and *Clostridium sensu stricto* prevent depression-like behavior via the gut microbiota [9]. This study aimed to examine whether physical activity is associated with reduced mild depression and altered gut microbiota composition in Japanese adult women.

## Materials and methods

Study design

This cross-sectional observational study was conducted at the Health Check-up Center of Kawasaki Medical School Hospital in Okayama, Japan. Study participants were enrolled between May and December 2022. The study participants were randomly selected women aged 50 to 80 who underwent a health check-up, except smokers and persons taking antibiotics and/or antidepressants.

In this study, the required total sample size was calculated to be n = 52 by using G*Power (Heinrich-Heine-Universität Düsseldorf, Düsseldorf, Germany) for an a priori power analysis with Cohen’s f = 0.4, α = 0.05, and power (1-β) = 0.80. In fact, the sample size was 48, which was almost equal to the required total sample size. Although this sample size slightly reduces the statistical power (to approximately 76%-78%), the study remains reasonably powered to detect the expected medium-to-large effect size. Additionally, this sample size is also consistent with prior research in cognitive and behavioral interventions among older adults, which satisfied both reliability and feasibility [10,11].

**Figure 1:**
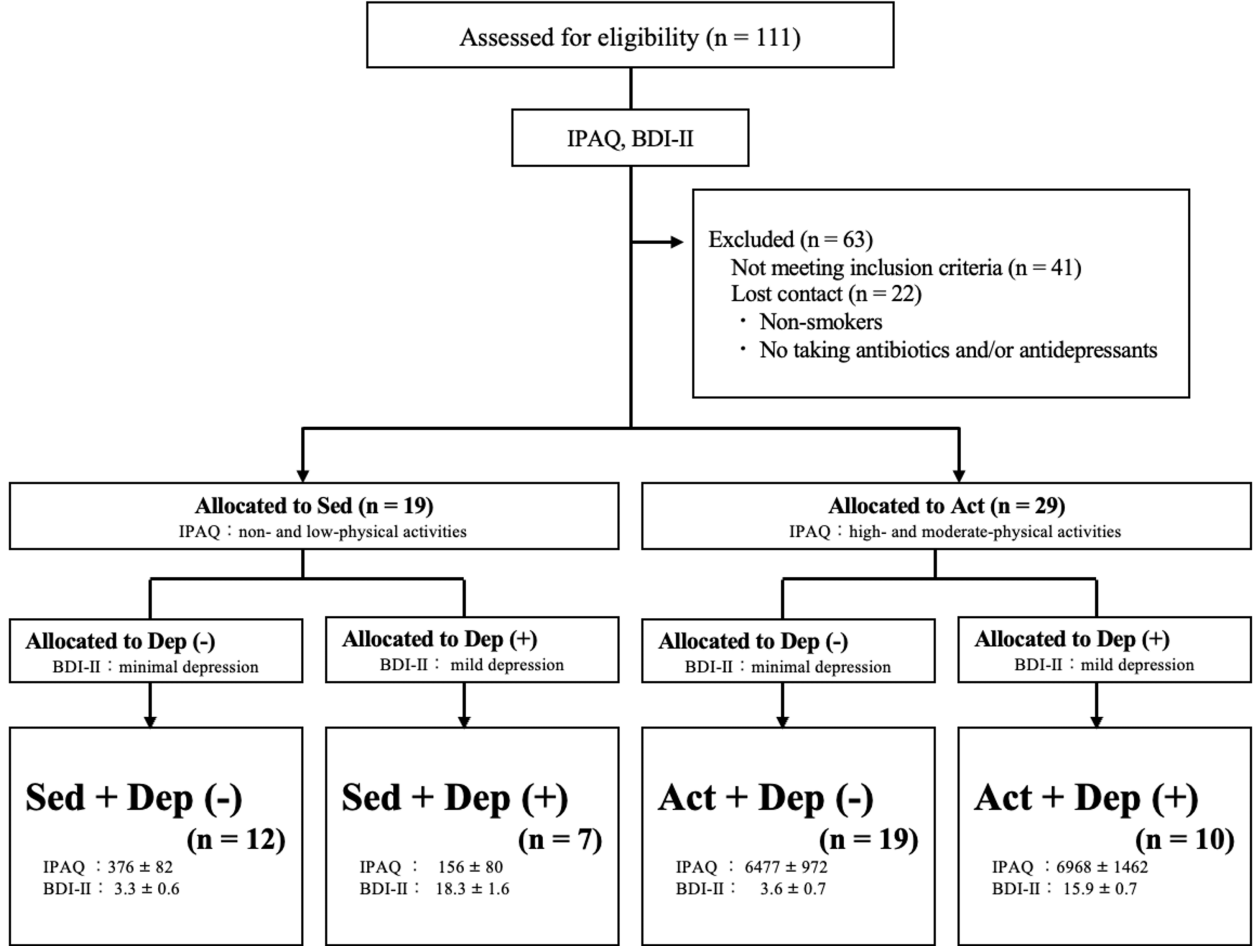
Flow diagram of participants IPAQ score and BDI-II score were expressed as the mean ± SE. Act participants: high physical activity level (one of the following criteria: (1) engaging in vigorous-intensity physical activity on a minimum of 3 day/wk, achieving a total of 1,500 MET-min/wk, or (2) engaging in combined walking and moderate- and/or vigorous-intensity activities at least 7 day/wk, accumulating 3,000 MET-min/wk) or moderate physical activity level (one of the criteria: (1) engaging in vigorous-intensity activity for at least 20 min/day on 3 or more day/wk, (2) engaging in moderate-intensity physical activity or walking for at least 30 min/day on at least 5 day/wk, or (3) engaging in combined walking and moderate- and/or vigorous-intensity activities on at least 5 day/wk, resulting in a total of at least 600 MET-min/wk). The participants who did not meet the criteria for either moderate or high levels of physical activity were classified as having a low or inactive level of physical activity, as the Sed group. Using the BDI-II cutoff score (≤13) based on established guidelines, participants were classified as no depression (Dep (-) group) or mild depression (Dep (+) group) MET: metabolic equivalent of task; BDI-II: Beck Depression Inventory-II; IPAQ: International Physical Activity Questionnaire; SE: standard error

The participants answered the International Physical Activity Questionnaire (IPAQ) to assess physical activity and the Beck Depression Inventory-II (BDI-II) to assess the symptoms of depression (Supplemental material 1). Firstly, they were divided into two groups: sedentary (Sed) and activity (Act) participants. The classification of physical activity levels was based on the IPAQ [12,13]. Namely, the Act group had a high or moderate physical activity level, and the Sed group had a low or no physical activity level. Moreover, each group was divided into two groups: low BDI-II score (≤13) [14-16] (minimal depression: Dep (-)) and the rest (mild depression: Dep (+)). Accordingly, in this study, there were four groups: Sed + Dep (-) (n = 12), Act + Dep (-) (n = 19), Sed + Dep (+) (n = 7), and Act + Dep (+) (n = 10) (Figure 1). The items measured were the gut microbiota, dietary questionnaire (Brief-Type Self-Administered Diet History Questionnaire (BDHQ) [17]) (Supplemental material 1), body weight, body fat percentage, blood pressure, and biochemical parameters (blood glucose, HbA1c, triglyceride (TG), high-density lipoprotein cholesterol (HDL-C), and low-density lipoprotein cholesterol (LDL-C)).

Analysis of the microbiota

All participants collected a stool sample at home. The collected samples, which were scraped on the surface of their stool after defecation, were taken into tubes containing guanidine thiocyanate solution (Techno Suruga Laboratory Co. Ltd., Shizuoka, Japan). The DNA preservation solution was stable for one month at room temperature for DNA collected from stool samples. After sample collection, within 48 hours and storage at 4°C until DNA extraction, DNA was subjected to amplicon sequence analysis using a next-generation sequencing (NGS) instrument, the MiSeq system (Illumina, San Diego, CA, US) by Techno Suruga Laboratory Co. Ltd. [18]. In brief, the extracted DNA using an automated DNA isolation system (Kurabo, Osaka, Japan) was amplified with the V3-V4 regions of 16S rRNA by the Pro341F/Pro805R primers and the dual-index method [18], and then was visualized by electrophoresis. Barcoded amplicons were paired-end sequenced using a 2 × 284 bp cycle and the MiSeq system with MiSeq Reagent Kit v3 (600 cycles). The paired-end sequencing read quality was checked by the FASTX-Toolkit as a collection of command line tools.

We analyzed the 16S rRNA sequences using Quantitative Insights into Microbial Ecology (QIIME) 2.0 v2023.2. DADA2 denoise-single plugin ver. 2017.6.0 with the default option was used for quality filtering and chimera sequence filtering [19]. To clear the taxonomy assignment, Silva database v138 was used based on an average percent identity of 99% [20]. Alpha-diversities (amplicon sequence variants (ASVs), Chao1, Shannon index, and Simpson index) were calculated. Beta-diversity was assessed using unweighted Unifrac distance metrics, and principal coordinates analysis (PCoA) was performed to calculate the pattern of differences.

Statistical analysis

Statistical calculations (including data visualization) were carried out with software R (ver. 4.4.1) (R Foundation for Statistical Computing, Vienna, Austria) and RStudio (ver. 2024.09.0+375) (Posit Software, Boston, MA, US). The comparison of means between groups was analyzed using a two-way analysis of variance (ANOVA), and then, the Dunn test was conducted as a post hoc test. p-value < 0.05 was considered to indicate statistical significance. The statistical distances among the four groups in the beta-diversity of the gut microbiota were analyzed using permutational multivariate ANOVA (PERMANOVA) with the vegan package, based on ASV-level Unifrac distances. To visualize the expression of the differentially expressed gut microbiota (species levels) for screening, we applied the volcano plot (cutoff: log2 fold change of 1 or higher, and cutoff: p-value = 0.05).

Ethics approval and informed consent

This study was carried out in accordance with the principles outlined in the Declaration of Helsinki. Also, this study was approved by the Ethics Committee of Kawasaki Medical School (5968-01), including Kawasaki University of Medical Welfare (23-005). Written informed consent was obtained from all participants.

## Results

The characteristics of the survey participants and biochemical parameters are presented in Table 1. No significant differences in participant characteristics were observed between the groups. The TG in the Sed + Dep (+) group was significantly lower than that in the Sed + Dep (−) group (p < 0.05, Table 1).

**Table 1:** Participant characteristics Data were expressed as the mean ± SE. Comparison of means between groups was analyzed using a two-way ANOVA, and then, the Dunn test was conducted as a post hoc test *p < 0.05 vs. Sed + Dep (-) SE: standard error; ANOVA: analysis of variance; HDL: high-density lipoprotein; LDL: low-density lipoprotein

	Sed + Dep (-) (n = 12)	Act + Dep (-) (n = 19)	Sed + Dep (+) (n = 7)	Act + Dep (+) (n = 10)	Activity	Depression	Interaction
Age (year)	59.9 ± 1.6	61.9 ± 2.1	57.3 ± 1.5	60.4 ± 1.5	F _(1, 47)_ = 1.2991, η²ₚ = 0.0287	F _(1, 47)_ = 0.8527, η²ₚ = 0.0190	F _(1, 47)_ = 0.0647, η²ₚ = 0.0015
Height (cm)	155.8 ± 1.0	154 ± 1.1	158.4 ± 2.1	156.5 ± 1.3	F _(1, 47)_ = 1.6030, η²ₚ = 0.0368	F _(1, 47)_ = 2.9566, η²ₚ = 0.0658	F _(1, 47)_ = 0.0017, η²ₚ = 0.0000
Body weight (kg)	54.8 ± 1.8	50.1 ± 2.1	57.2 ± 5.6	55.9 ± 2.4	F _(1, 47)_ = 0.9658, η²ₚ = 0.0225	F _(1, 47)_ = 1.8082, η²ₚ = 0.0413	F _(1, 47)_ = 0.3004, η²ₚ = 0.0071
Body fat (%)	30.8 ± 1.5	27.0 ± 2.0	31.0 ± 4.7	31.0 ± 1.9	F _(1, 47)_ = 0.5160, η²ₚ = 0.0124	F _(1, 47)_ = 0.5893, η²ₚ = 0.0142	F _(1, 47)_ = 0.5194, η²ₚ = 0.0125
Systolic blood pressure (mmHg)	125.5 ± 4.5	124.7 ± 4.1	131.7 ± 4.2	126.8 ± 4.5	F _(1, 47)_ = 0.3082, η²ₚ = 0.0073	F _(1, 47)_ = 0.6604, η²ₚ = 0.0155	F _(1, 47)_ = 0.1643, η²ₚ = 0.0039
Diastolic blood pressure (mmHg)	79.7 ± 2.9	77.1 ± 2.8	83.6 ± 3.2	78.6 ± 2.1	F _(1, 47)_ = 1.3049, η²ₚ = 0.0301	F _(1, 47)_ = 0.6308, η²ₚ = 0.0148	F _(1, 47)_ = 0.1209, η²ₚ = 0.0029
Blood glucose (mg/dL)	97.5 ± 1.7	101.1 ± 3.7	105.9 ± 5.7	95.1 ± 3.3	F _(1, 47)_ = 0.6986, η²ₚ = 0.0168	F _(1, 47)_ = 0.0802, η²ₚ = 0.0020	F _(1, 47)_ = 2.8833, η²ₚ = 0.0657
HbA1c (%)	5.7 ± 0.1	5.9 ± 0.2	5.9 ± 0.0	5.8 ± 0.0	F _(1, 47)_ = 0.1580, η²ₚ = 0.0054	F _(1, 47)_ = 0.0300, η²ₚ = 0.0010	F _(1, 47) _= 0.4104, η²ₚ = 0.0140
Triglyceride (mg/dL)	101.5 ± 9.7	83.6 ± 9.8	65.3 ± 4.6*	122.3 ± 30.7	F _(1, 47)_ = 1.3081, η²ₚ = 0.0309	F _(1, 47)_ = 0.0054, η²ₚ = 0.0001	F _(1, 47)_ = 4.8283, η²ₚ = 0.1054
HDL cholesterol (mg/dL)	61.6 ± 5.1	70.3 ± 3.0	78.4 ± 6.3	70.7 ± 5.5	F _(1, 47)_ = 0.0086, η²ₚ = 0.0002	F _(1, 47)_ = 2.8851, η²ₚ = 0.0657	F _(1, 47)_ = 2.6649, η²ₚ = 0.0610
LDL cholesterol (mg/dL)	139.9 ± 8.0	115.8 ± 8.3	110.7 ± 6.0	125.9 ± 11.3	F _(1, 47)_ = 0.1946, η²ₚ = 0.0047	F _(1, 47)_ = 0.8835, η²ₚ = 0.0211	F _(1, 47)_ = 3.7481, η²ₚ = 0.0838

The results of the dietary questionnaire BDHQ showed that carbohydrate consumption in the Dep (+) group was significantly higher than that in the Dep (-) group in Act participants (p < 0.001, Table 2). No differences, however, were observed in the other dietary parameters (Table 2).

**Table 2:** Comparison of energy and nutrient intakes according to BDHQ by each group Data were expressed as the mean ± SE. Comparison of means between groups was analyzed using a two-way ANOVA, and then, the Dunn test was conducted as a post hoc test ***p < 0.001 vs. Act + Dep (-) BDHQ: Brief-Type Self-Administered Diet History Questionnaire; SE: standard error; ANOVA: analysis of variance

	Sed + Dep (-) (n = 12)	Act + Dep (-) (n = 19)	Sed + Dep (+) (n = 7)	Act + Dep (+) (n = 10)	Activity	Depression	Interaction
Energy (kcal/day)	1,572.6 ± 100.0	1,498.9 ± 99.3	1,518.5 ± 123.8	1,935.0 ± 146.0	F _(1, 47)_ = 1.7346, η²ₚ = 0.0379	F _(1, 47)_ = 2.1542, η²ₚ = 0.0467	F _(1, 47)_ = 3.5473, η²ₚ = 0.0746
Protein (g/day)	64.9 ± 6.3	62.9 ± 6.1	56.5 ± 6.4	72.9 ± 7.7	F _(1, 47)_ = 0.9153, η²ₚ = 0.0204	F _(1, 47)_ = 0.0103, η²ₚ = 0.0002	F _(1, 47)_ = 1.4779, η²ₚ = 0.0325
Lipid (g/day)	48.3 ± 3.5	50.1 ± 4.0	49.1 ± 5.9	59.1 ± 4.6	F _(1, 47)_ = 1.4850, η²ₚ = 0.0326	F _(1, 47)_ = 1.0276, η²ₚ = 0.0228	F _(1, 47)_ = 0.7030, η²ₚ = 0.0157
Carbohydrates (g/day)	215.4 ± 15.2	186.9 ± 11.6	206 ± 20.8	270.6 ± 23.5***	F _(1, 47)_ = 0.9657, η²ₚ = 0.0215	F _(1, 47)_ = 4.0673, η²ₚ = 0.0846	F _(1, 47)_ = 6.3791, η²ₚ = 0.1266
Soluble dietary fiber (g/day)	3.3 ± 0.4	3.0 ± 0.3	2.4 ± 0.4	3.1 ± 0.5	F _(1, 47)_ = 0.2269, η²ₚ = 0.0051	F _(1, 47)_ = 0.8817, η²ₚ = 0.0196	F _(1, 47)_ = 1.9885, η²ₚ = 0.0432
Insoluble dietary fiber (g/day)	8.9 ± 0.9	7.9 ± 0.8	6.5 ± 0.9	8.9 ± 1.1	F _(1, 47)_ = 0.4890, η²ₚ = 0.0110	F _(1, 47)_ = 0.4558, η²ₚ = 0.0103	F _(1, 47)_ = 2.8747, η²ₚ = 0.0613
Dietary fiber (g/day)	12.6 ± 1.3	11.2 ± 1.1	9.3 ± 1.2	12.5 ± 1.7	F _(1, 47)_ = 0.4046, η²ₚ = 0.0091	F _(1, 47)_ = 0.4910, η²ₚ = 0.0110	F _(1, 47)_ = 2.4897, η²ₚ = 0.0536

**Figure 2:**
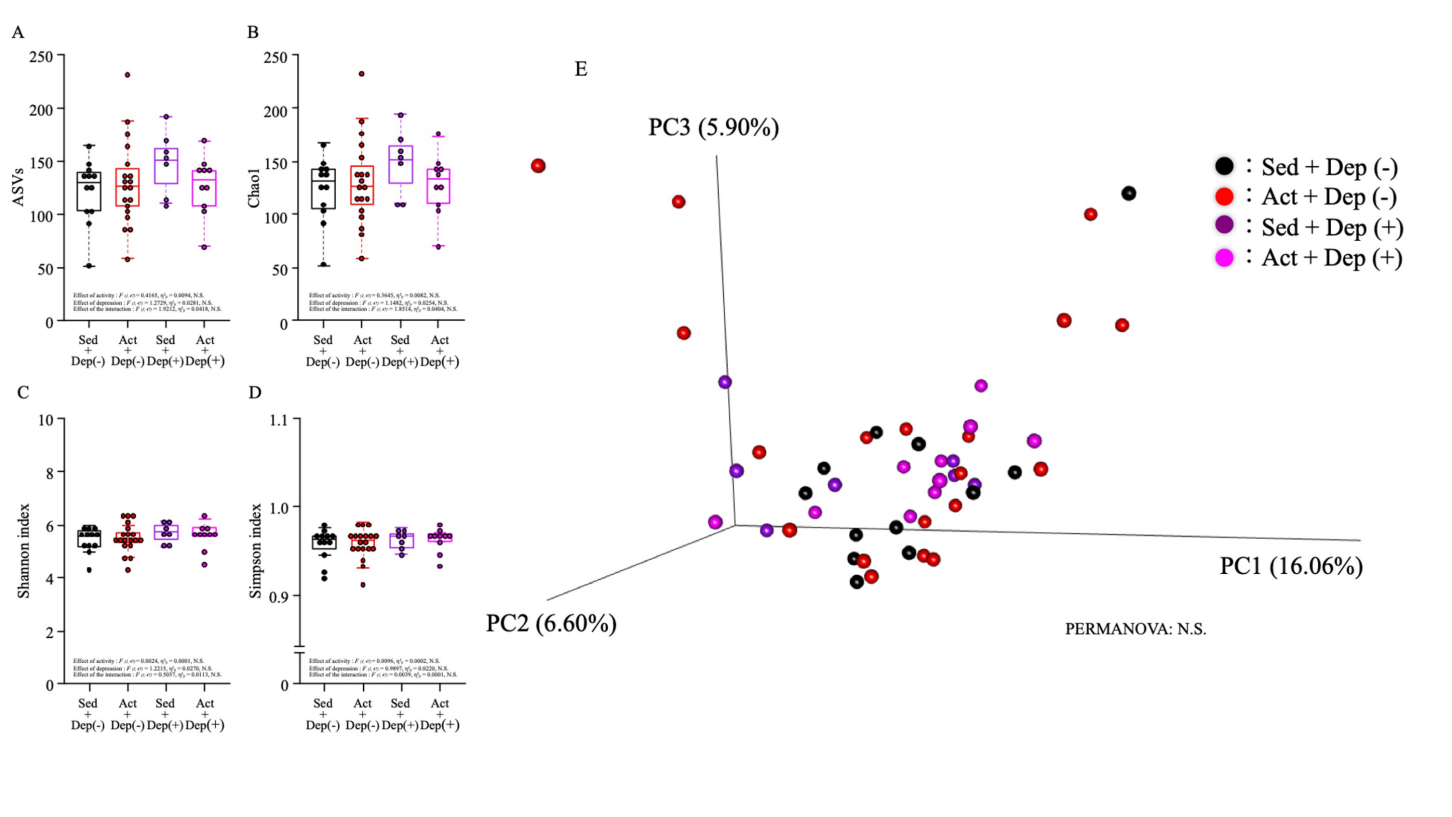
Bacterial alpha-diversity indexes ((A) ASVs, (B) Chao1, (C) Shannon index, and (D) Simpson index) and (E) beta-diversity index in each group Data were shown as beeswarm and box plots. Comparison of means between groups was analyzed using a two-way ANOVA, and then, the Dunn test was conducted as a post hoc test ASV: amplicon sequence variant; ANOVA: analysis of variance; PERMANOVA: permutational multivariate analysis of variance

Gut microbiota was evaluated for alpha- and beta-diversity (Figure 2). No significant differences were found with regard to alpha-diversities: ASVs, Chao1, Shannon index, and Simpson index. Also, there were no significant differences among the four groups in beta-diversity analyzed by PCoA.

**Figure 3:**
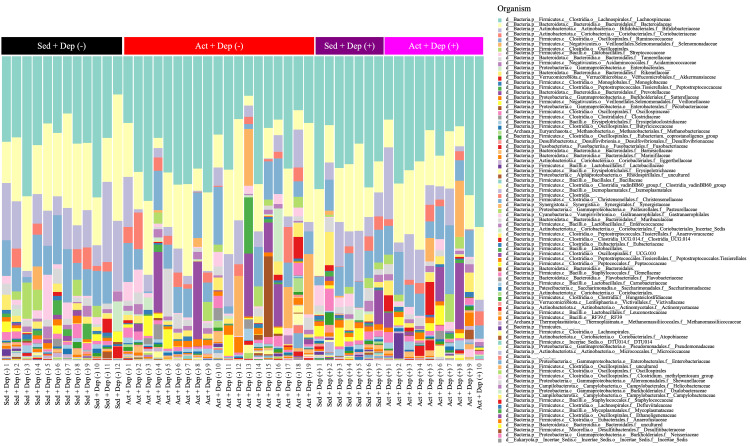
Taxonomy family of microbiota in feces

**Figure 4:**
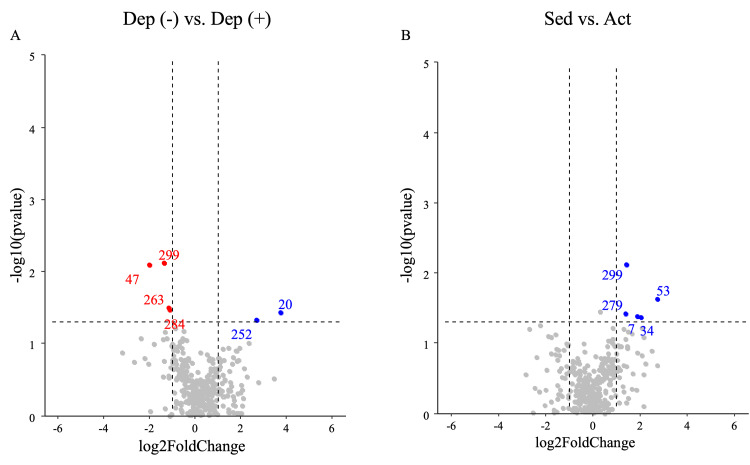
The differentiality expressed in the gut microbiota analyzed by volcano plots between every two groups: (A) Dep (-) vs. Dep (+); (B) Sed vs. Act Red and blue dots indicate that the bacterial abundances were significantly elevated/depressed for each group (p < 0.05, respectively). Dots in gray show unchanged abundances of bacteria

The proportion of relative abundances of gut microbiota at the family level in each sample is shown in Figure 3 as stacked bar plots. In addition, after the volcano plot analysis of the comparison between the Dep (-) group and the Dep (+) group, six bacteria (*Tyzzerella*_sp., *Faecalicoccus pleomorphus*, *Gemella*, *Holdemania filiformis*, *Eubacterium hallii* group, and DTU089) (Figure 4A) were extracted. Among these, *Tyzzerella*_sp., *Faecalicoccus pleomorphus*, *Gemella*, and *H. filiformis* were more abundant in the Dep (-) group, while *E. hallii* group and DTU089 were more abundant in the Dep (+) group. Moreover, between the Sed group and the Act group, five bacteria (*Megamonas*, *Prevotella*, *Catenibacterium*, *Family XIII AD3011 group*, and *H. filiformis*) (Figure 4B) were extracted. Please see appendices.

## Discussion

In this study, to evaluate the relationship between depression symptoms and physical activity within the changes in the gut microbiota in adult women, participants were divided by depression score and physical activity, and the characteristics of the gut microbiota of each group were analyzed. Although the levels of alpha- and beta-diversities in the gut microbiota were not different among the four groups, we found that the gut microbiota changed under the influence of mild depression and physical activity.

In this study, low TG concentration in the plasma of the Sed + Dep (+) group was observed. Thus, the TG level, along with the HDL cholesterol level, was within the normal range (<150 mg/dL) of the diagnostic criteria for metabolic syndrome in Japanese women, despite Dep (+).

It was reported that exercise showed higher alpha-diversity in the gut microbiota [21], while patients with MDD found no significant difference in alpha-diversity compared with healthy controls [22]. The participants in this study were taken from the general adult woman population, suggesting no engagement in daily physical activities like athletes. Thus, no effect of physical activity on the diversity of the gut microbiota was observed.

Although in this study we first conducted linear discriminant analysis effect size (LEfSe) analysis, no characteristic differences were found between groups at each taxonomic level (Supplemental material 3). As no notable phylogenetic differences were observed between the groups, we conducted further analysis at the species level. The participants were examined for changes in gut microbes based on comparisons of groups divided by physical activity and mild depression characteristics, with no differences in physical characteristics. In a comparison of mild depressive symptoms, we detected significantly more presence of *E. hallii* and DTU089 in the Dep (+) group than in the Dep (-) group. *Eubacterium* has been reported to have lower levels in patients with MDD compared to healthy controls [23] and to be negatively correlated with serum brain-derived neurotrophic factor (BDNF) levels [24]. Therefore, it is possible that *E. hallii* may have influenced the elevated BDI-II score.

In a previous study analyzing the gut microbiota of professional and amateur cyclists, a higher percentage of *Prevotella* spp. was reported in response to training volume [25]. In this study, *Prevotella* spp. were significantly higher in the moderately and highly physically active groups, suggesting that the bacteria may be increased by exercise. Interestingly, a higher prevalence of *Prevotella* was observed in individuals reporting higher intake of carbohydrates and simple sugars [26]. Thus, it is possible that the high abundance of *Prevotella* is related to high physical activity.

**Figure 5:**
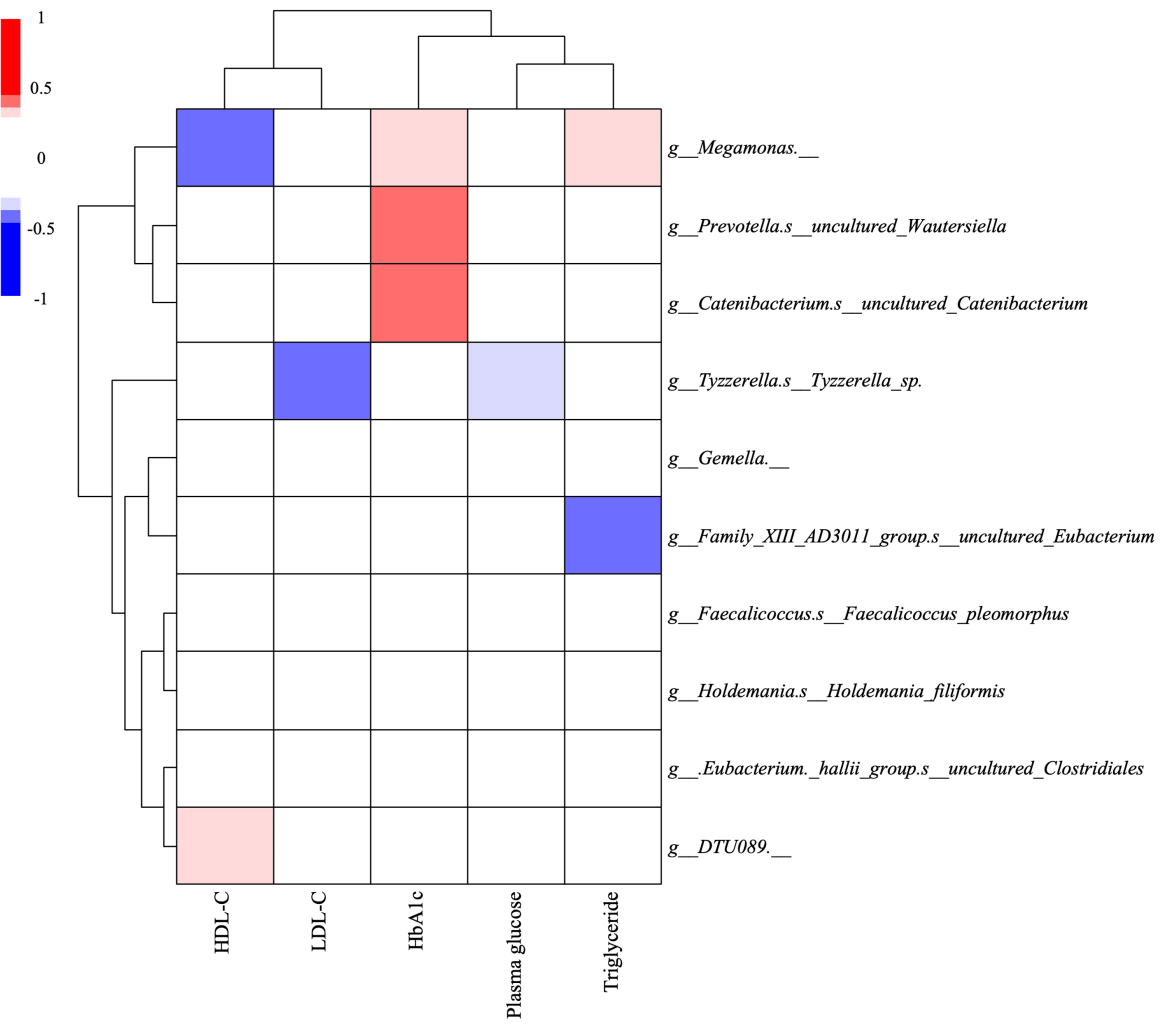
The heat map of correlations between biochemical parameters and gut microbiota identified as differentially abundant through volcano plot analysis (Figure 4) The darker red bar indicates a strongly positive correlation (toward r = 1.0), while the darker blue bar indicates a strongly negative correlation (toward r = −1.0). The represented white regions are not significant at p ≥ 0.05 HDL-C: high-density lipoprotein cholesterol; LDL-C: low-density lipoprotein cholesterol

The heat map analysis of the correlation between the extracted gut microbiota and the participants’ blood test results is shown below (Figure 5). *Megamonas* was shown to have a significant positive correlation with serum TGs and HbA1c. The genus *Megamonas* has been reported to be more prevalent in obese and type 2 diabetic patients and to be positively correlated with TG and HbA1c, suggesting an association with lifestyle-related diseases [27]. In contrast, a recent study comparing the intestinal microbiota among athletes, individuals with high physical activity, and those with low physical activity found that *Megamonas* abundance increased with higher physical activity levels, which is consistent with the findings of the present study [28]. A recent study reported that in spinal cord injury patients with severely restricted physical activity, *Megamonas* levels are decreased along with decreased acetate and propionate levels in SCFAs, which were the main metabolites generated [29]. In fact, *Megamonas* produces acetate and propionate based on glucose in vitro [30].

Unfortunately, a systematic review on depression and *Megamonas* reported that patients with MDD have more *Megamonas*, whereas another paper reported less *Megamonas* in late-life depression [31]. Thus, the association between depression and *Megamonas* is not consistent. In addition, we observed positive and/or negative correlation between each bacterium and biochemical parameter. In fact, it is known that *Tyzzerella* has a function in the fermentation of dietary fibers to produce SCFAs [32] and is associated with regulating lipid metabolism [33]. Also, *Clostridiales bacterium* DTU089 was more abundant in participants with lower protein intake [34]. It was suggested that *Catenibacterium* has the ability to synthesize SCFAs, associated with high HbA1c levels [35]. Therefore, our results were supported by these studies. However, the detailed mechanism remains unclear.

On the other hand, in this study, the richness of relative abundance of *H. filiformis* was common between the Act and Dep (-) groups. It has been reported that *Holdemania* is a more common bacterium in patients with MDD and depression [36]. A recent study in Japanese subjects, however, suggested that *Holdemania* suppresses depression [5]. *Holdemania* is a Gram-positive anaerobic genus from the family Erysipelotrichaceae in the phylum Firmicutes and is a glycolytic bacterium [37]. *Holdemania* may have increased in Act + Dep (+) due to higher carbohydrate intake than in Act + Dep (-). The abundances of *Megamonas*, *Prevotella*, and *Catenibacterium* were high in the exercise group, showing a positive correlation with HbA1c. Thus, these relationships may indicate changes in the gut microbiota related to excessive carbohydrate intake in the Act + Dep (+) group. However, *Holdemania* is the only bacterium common to both Act and Dep (-), and it was extracted as the bacterium suggesting a relationship between exercise and the prevention of depression independent of blood parameters.

In this study, we found results suggesting differences in the composition of the gut microbiota in Japanese adult women, indicating a correlation between physical activity and mild depression. The findings in this study appear to demonstrate the health benefits of exercise for Japanese adult women who are exposed to various stresses on a daily basis. It is expected that further detailed intensity, duration, frequency, and type of exercise habits will clarify the relationship between the preventive effects of exercise on depression and the gut microbiota.

There are limitations to this study. The main limitation is that this study has a cross-sectional design. Further longitudinal studies are needed to clarify causal relationships. This study included only Japanese participants, which may limit the generalizability of the results. We believe that a more comprehensive understanding of the relationship between gut microbiota and depression and physical activity can be obtained by combining the findings of this study with those of previous studies in other ethnic groups. Although the overall sample size (n = 48) approached the estimated requirement (n = 52), the size of some subgroups (ranging from n = 7 to n = 19) was relatively small. This limitation may have reduced the statistical power to detect group differences, increasing the risk of type II error. Future studies with larger and more balanced subgroup sizes are warranted to confirm the stability and generalizability of our findings. Although carbohydrate intake showed a statistically significant interaction, the detailed effects of dietary components on gut microbiota were beyond the scope of the present study and should be addressed in future research. Finally, this study recruited participants who were relatively healthy and able to attend health screening centers on their own. This may have introduced bias during the sample collection phase, which may affect the generalizability of the results.

## Conclusions

This study aimed to examine whether physical activity is associated with reduced mild depression and altered gut microbiota composition in Japanese adult women. Although no clear effect of mild depression or physical activity on gut microbiota diversity was observed, the results suggest a possible involvement of *H. filiformis* in the association between physical activity and depression prevention. These results are fundamental data for future improvement of depression prevention and exercise intervention against depression.

## Figures and Tables

**Figure 6:**
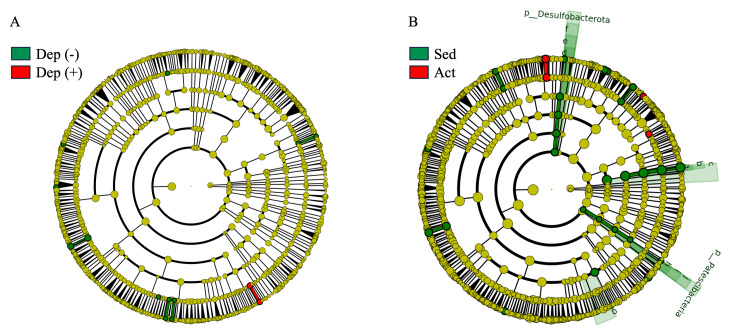
Gut microbiota identified by LEfSe: (A) Dep (-) vs. Dep (+); (B) Sed vs. Act LEfSe: linear discriminant analysis effect size

## Appendices

Supplemental material 1

**Table 3:** Summary of assessment tools for depression, physical activity, and dietary intake BDI-II: Beck Depression Inventory-II; IPAQ: International Physical Activity Questionnaire; BDHQ: Brief-Type Self-Administered Diet History Questionnaire; MET: metabolic equivalent of task

Item	BDI-II	IPAQ	BDHQ
Target of evaluation	Severity of depressive symptoms	Physical activity (exercise and daily activity)	Dietary intake frequency and nutrient intake
Number of items	21 items	7 items	Approximately 75 items
Question format	Self-administered; 4-point Likert scale (0–3)	Self-administered/interview; multiple activity-related choices	Self-administered checklist
Score range	0–63 points	Classified by METs (intensity × time)	Estimated intake of nutrients and food groups (e.g., energy and vitamins)
Score interpretation	Minimal: 0–13, mild: 14–19, moderate: 20–28, severe: 29–63	Low, moderate, high	Enables comparison with dietary reference intakes
References	Kojima et al. [14]; Dozois et al. [15]	Craig et al. [12]; Murase et al. [38]	Kobayashi et al. [17]

Supplemental material 2 

**Table 4:** The code list of gut microbiota in the volcano plots (Figure 4)

Code	Phylum	Class	Order	Family	Genus	Species
7	p__Firmicutes	c__Negativicutes	o__Veillonellales-Selenomonadales	f__Selenomonadaceae	g__Megamonas	__
20	p__Firmicutes	c__Clostridia	o__Lachnospirales	f__Lachnospiraceae	g__[Eubacterium]_hallii_group	s__uncultured_Clostridiales
34	p__Bacteroidota	c__Bacteroidia	o__Bacteroidales	f__Prevotellaceae	g__Prevotella	s__uncultured_Wautersiella
47	p__Firmicutes	c__Clostridia	o__Lachnospirales	f__Lachnospiraceae	g__Tyzzerella	s__Tyzzerella_sp.
53	p__Firmicutes	c__Bacilli	o__Erysipelotrichales	f__Erysipelatoclostridiaceae	g__Catenibacterium	s__uncultured_Catenibacterium
252	p__Firmicutes	c__Clostridia	o__Oscillospirales	f__Ruminococcaceae	g__DTU089	__
263	p__Firmicutes	c__Bacilli	o__Erysipelotrichales	f__Erysipelotrichaceae	g__Faecalicoccus	s__Faecalicoccus_pleomorphus
279	p__Firmicutes	c__Clostridia	o__Peptostreptococcales-Tissierellales	f__Anaerovoracaceae	g__Family_XIII_AD3011_group	s__uncultured_Eubacterium
284	p__Firmicutes	c__Bacilli	o__Staphylococcales	f__Gemellaceae	g__Gemella	__
299	p__Firmicutes	c__Bacilli	o__Erysipelotrichales	f__Erysipelotrichaceae	g__Holdemania	s__Holdemania_filiformis

Supplemental material 3 

## References

[1] (2024). Global Burden of Disease. https://vizhub.healthdata.org/gbd-results/.

[2] GBD 2017 Disease and Injury Incidence and Prevalence Collaborators (2018). Global, regional, and national incidence, prevalence, and years lived with disability for 354 diseases and injuries for 195 countries and territories, 1990-2017: a systematic analysis for the Global Burden of Disease Study 2017. Lancet.

[3] Salk RH, Hyde JS, Abramson LY (2017). Gender differences in depression in representative national samples: meta-analyses of diagnoses and symptoms. Psychol Bull.

[4] Dowlati Y, Herrmann N, Swardfager W, Liu H, Sham L, Reim EK, Lanctôt KL (2010). A meta-analysis of cytokines in major depression. Biol Psychiatry.

[5] Yang Y, Mori M, Wai KM (2023). The association between gut microbiota and depression in the Japanese population. Microorganisms.

[6] Mikkelsen K, Stojanovska L, Polenakovic M, Bosevski M, Apostolopoulos V (2017). Exercise and mental health. Maturitas.

[7] Cryan JF, Dinan TG (2012). Mind-altering microorganisms: the impact of the gut microbiota on brain and behaviour. Nat Rev Neurosci.

[8] Allen JM, Mailing LJ, Niemiro GM (2018). Exercise alters gut microbiota composition and function in lean and obese humans. Med Sci Sports Exerc.

[9] Watanabe C, Oyanagi E, Aoki T (2023). Antidepressant properties of voluntary exercise mediated by gut microbiota. Biosci Biotechnol Biochem.

[10] Ranawat A, Yadav D, Solanki RK (2014). A comparative study of TSH and lipid profile in depressed postmenopausal women. Int J Sci Appl Res.

[11] Draghici R, Opris S, Rusu A (2022). The link between lipid profile, cardiovascular risk and mood disorders appearance in older patients. J Gerontol Geriatr Med.

[12] Craig CL, Marshall AL, Sjöström M (2003). International physical activity questionnaire: 12-country reliability and validity. Med Sci Sports Exerc.

[13] Tomioka K, Iwamoto J, Saeki K, Okamoto N (2011). Reliability and validity of the International Physical Activity Questionnaire (IPAQ) in elderly adults: the Fujiwara-kyo study. J Epidemiol.

[14] Kojima M, Furukawa T, Takahashi H, Kawai M, Nagaya T, Tokudome S (2002). Cross-cultural validation of the Beck Depression Inventory-II in Japan. Psychiatry Res.

[15] Dozois DJ, Dobson KS, Ahnberg JL (1998). A psychometric evaluation of the Beck Depression Inventory-II. Psychol Assess.

[16] Beck A, Steer R, Brown G (1996). Manual for the Beck Depression Inventory-Ⅱ. San Antoniox The Psychological Cooporation.

[17] Kobayashi S, Murakami K, Sasaki S (2011). Comparison of relative validity of food group intakes estimated by comprehensive and brief-type self-administered diet history questionnaires against 16 d dietary records in Japanese adults. Public Health Nutr.

[18] Takahashi S, Tomita J, Nishioka K, Hisada T, Nishijima M (2014). Development of a prokaryotic universal primer for simultaneous analysis of Bacteria and Archaea using next-generation sequencing. PLoS One.

[19] Callahan BJ, McMurdie PJ, Rosen MJ, Han AW, Johnson AJ, Holmes SP (2016). DADA2: high-resolution sample inference from Illumina amplicon data. Nat Methods.

[20] Quast C, Pruesse E, Yilmaz P (2013). The SILVA ribosomal RNA gene database project: improved data processing and web-based tools. Nucleic Acids Res.

[21] Clarke SF, Murphy EF, O'Sullivan O (2014). Exercise and associated dietary extremes impact on gut microbial diversity. Gut.

[22] Sanada K, Nakajima S, Kurokawa S (2020). Gut microbiota and major depressive disorder: a systematic review and meta-analysis. J Affect Disord.

[23] Yang J, Zheng P, Li Y (2020). Landscapes of bacterial and metabolic signatures and their interaction in major depressive disorders. Sci Adv.

[24] Kim CS, Cha L, Sim M, Jung S, Chun WY, Baik HW, Shin DM (2021). Probiotic supplementation improves cognitive function and mood with changes in gut microbiota in community-dwelling older adults: a randomized, double-blind, placebo-controlled, multicenter trial. J Gerontol A Biol Sci Med Sci.

[25] Petersen LM, Bautista EJ, Nguyen H (2017). Community characteristics of the gut microbiomes of competitive cyclists. Microbiome.

[26] Wu GD, Chen J, Hoffmann C (2011). Linking long-term dietary patterns with gut microbial enterotypes. Science.

[27] Lauw S, Kei N, Chan PL (2023). Effects of synbiotic supplementation on metabolic syndrome traits and gut microbial profile among overweight and obese Hong Kong Chinese individuals: a randomized trial. Nutrients.

[28] Xu Y, Zhong F, Zheng X, Lai HY, Wu C, Huang C (2022). Disparity of gut microbiota composition among elite athletes and young adults with different physical activity independent of dietary status: a matching study. Front Nutr.

[29] Jing Y, Yang D, Bai F (2023). Spinal cord injury-induced gut dysbiosis influences neurological recovery partly through short-chain fatty acids. NPJ Biofilms Microbiomes.

[30] Hayashi T, Yamashita T, Watanabe H (2018). Gut microbiome and plasma microbiome-related metabolites in patients with decompensated and compensated heart failure. Circ J.

[31] Chen Y, Le D, Xu J, Jin P, Zhang Y, Liao Z (2024). Gut microbiota dysbiosis and inflammation dysfunction in late-life depression: an observational cross-sectional analysis. Neuropsychiatr Dis Treat.

[32] Xu Y, Yang Y, Li B, Xie Y, Shi Y, Le G (2022). Dietary methionine restriction improves gut microbiota composition and prevents cognitive impairment in D-galactose-induced aging mice. Food Funct.

[33] Wang J, Hu Q, Wang J (2025). Role of gut microbiota and fecal metabolites in the protective effect of soybean pulp-rich diet against estrogen-induced cholestasis in rats. Curr Res Food Sci.

[34] Farsijani S, Cauley JA, Peddada SD (2023). Relation between dietary protein intake and gut microbiome composition in community-dwelling older men: findings from the Osteoporotic Fractures in Men Study (MrOS). J Nutr.

[35] Mitrović M, Stanković Popović V, Erceg S (2025). Exploring the potential of oral butyrate supplementation in metabolic dysfunction-associated steatotic liver disease: subgroup insights from an interventional study. Int J Mol Sci.

[36] Gao M, Wang J, Liu P (2023). Gut microbiota composition in depressive disorder: a systematic review, meta-analysis, and meta-regression. Transl Psychiatry.

[37] Raimondi S, Musmeci E, Candeliere F, Amaretti A, Rossi M (2021). Identification of mucin degraders of the human gut microbiota. Sci Rep.

[38] Murase N, Katsumura T, Ueda C, Inoue S, Shimomitsu T (2002). Validity and reliability of Japanese version of International Physical Activity Questionnaire. J Health Welfare Stat.

